# *FGFR2* molecular analysis and related clinical findings in one Chinese family with Crouzon Syndrome

**Published:** 2012-02-12

**Authors:** Ying Lin, Xuanwei Liang, Siming Ai, Chuan Chen, Xialin Liu, Lixia Luo, Shaobi Ye, Baoxin Li, Yizhi Liu, Huasheng Yang

**Affiliations:** 1 State Key Laboratory of Ophthalmology, Zhongshan Ophthalmic Center, Sun Yat-sen University, Guangzhou, China; 2 Department of Pharmacology (State-Province Key Laboratories of Biomedicine-Pharmaceutics of China), Harbin Medical University, Harbin, Heilongjiang, China

## Abstract

**Purpose:**

The purposed of this study was to investigate the fibroblast growth factor receptor 2 (*FGFR2*) gene in one Chinese family with Crouzon syndrome and to characterize the related clinical features.

**Methods:**

One family underwent complete ophthalmic examinations, and two patients were diagnosed with Crouzon syndrome. Genomic DNA was extracted from leukocytes of peripheral blood collected from the family and 100 unrelated control subjects from the same population. Exons 8 and 10 of *FGFR2* were amplified by polymerase chain reaction (PCR) and directly sequenced. We performed ophthalmic examinations, including best-corrected visual acuity, slit-lamp examination, fundus examination, Pentacam, Goldmann perimetry, and computed tomography (CT) of the skull.

**Results:**

The two patients were affected with shallow orbits and ocular proptosis, accompanied by midface hypoplasia, craniosynostosis, and clinically normal hands and feet. A heterozygous *FGFR2* missense mutation c.866A>C (Gln289Pro) in exon 8 was identified in the affected individuals, but not in any of the unaffected family members and the normal controls.

**Conclusions:**

Although *FGFR2* mutations and polymorphisms have been reported in various ethnic groups, especially in the area of osteology, we report, for the first time, the identification of one new *FGFR2* mutation in Chinese patients with Crouzon syndrome.

## Introduction

Crouzon syndrome (CS), the most common craniosynostosis syndrome, is an autosomal-dominant inherited disorder, characterized by craniosynostosis, shallow orbits, ocular proptosis, midface hypoplasia, and a curved, beaklike nose [1-4].

Although this disorder is caused by the premature fusion of cranial sutures, leading to the clinical condition of craniosynostosis, which falls under the category of osteology, many patients would choose ophthalmology, due to the ocular proptosis and exposure keratitis. Until now, it was known that craniosynostosis is related to the fibroblast growth factor receptors (FGFRs).

Fibroblast growth factors (FGFs) and their receptors (FGFRs) constitute an elaborate signaling system that is involved in the developmental processes of most mammalian tissues. FGFRs are transmembrane proteins consisting of an extracellular ligand-binding domain and composed of three immunoglobulin (Ig)-like domains, a transmembrane domain, and an intracellular domain, which carries tyrosine kinase activity. Ligand-binding specificity of FGFRs depends on the third extracellular Ig-like domain, which is subject to alternative splicing, which generates a variety of receptor isoforms. Three different splice variants, IgIIIa, IgIIIb, and IgIIIc, have been identified [5,6].

It is known that Crouzon syndrome is usually caused by mutations in the *FGFR2* gene located on chromosome 10q26. Over 50 different mutations have been described in Crouzon syndrome [1,7-12], with approximately 95% of cases having mutations in just two exons of the gene, IIIa (8) and IIIc (10), which encode the extracellular immunoglobulin-like III (IgIII) domain of the protein [13].

This study reported the mutational analysis of one Chinese family with Crouzon syndrome at the gene level, along with related clinical features, and identified one heterozygous mutation.

## Methods

### The Crouzon syndrome family

**Figure 1:**
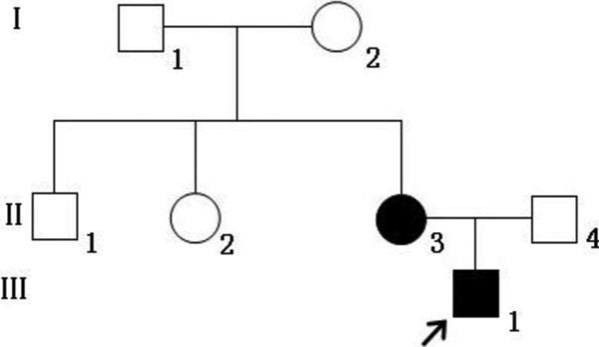
The pedigree of a Chinese family with Crouzon syndrome. Squares denote males, and circles denote females. The shaded symbols indicate ophthalmologist-confirmed Crouzon syndrome. The arrow points to the proband.

Two patients in one Chinese family (Figure 1) were diagnosed as having Crouzon syndrome at the Zhongshan Ophthalmic Center. We performed ophthalmic examinations, as follows: Visual acuity was examined using the ETDRS chart (Precision Vision, La Salle, IL). An anterior segment photograph was obtained using a BX 900 Slit Lamp (Haag-Streit, Bern, Switzerland). Anterior segment measurements were measured with Pentacam HR version 70700 (Oculus, Wetzlar, Germany). In addition, computed tomography (CT) and physical examinations were performed to exclude systemic diseases.

### Sample collection

The affected family was identified at the Zhongshan Ophthalmic Center. One hundred subjects without diagnostic features of Crouzon syndrome, from the same population, were recruited to serve as normal controls. After informed consent was obtained from all participating individuals, following the principles of the Declaration of Helsinki, venous blood samples were collected for genomic DNA extraction from peripheral blood leucocytes, using standard protocols.

### Mutation detection

Exons 8 and 10 of the *FGFR2* gene were amplified by polymerase chain reaction (PCR) with primers [14] (Table 1). Briefly, PCR was conducted in 50-μl reactions. The cycling profile included one cycle at 94 °C for 5 min, followed by 40 cycles at 94 °C for 45 s, 52–66 °C for 45 s, and 72 °C for 45 s, as well as one cycle at 72 °C for 10 min. The PCR products were sequenced from both directions with an ABI3730 Automated Sequencer (PE Biosystems, Foster City, CA). The sequencing results were analyzed using Chromas (version 2.3; Technelysium Pty Ltd, Brisbane, QLD, Australia), and they were compared with the reference sequences in the database at the National Center for Biotechnology Information (NCBI NC_000010).

**Table 1:** Primers used for PCR.

**Exon**	**Forward (5′-3′)**	**Reverse (5′-3′)**	**Product size (bp)**	**Annealing temperature (°C)**
*FGFR2-8 (IIIa)*	GGTCTCTCATTCTCCCATCCC	CCAACAGGAAATCAAAGAACC	325	61
*FGFR2-10 (IIIc)*	CCTCCACAATCATTCCTGTGTC	ATAGCAGTCAACCAAGAAAAGGG	257	61

Summary of the primers and products length used for the amplification of the exons of* FGFR 2*.

The superimposed mutant PCR products were subcloned into pGEM-T vector (Promega, Madison, WI) and sequenced to identify the mutation. Briefly, PCR products were purified by gel extraction, using gel extraction kits (Axygen, Union City, CA) according to the manufacturer's instructions. The purified PCR fragments were ligated into the pGEM-T easy vector (Invitrogen, Carlsbad, CA), and the resulting plasmids were transfected, by heat shock, into DH5a-competent *Escherichia coli* for propagation. The plasmid DNA was sequenced using the ABI3700 and the T7 primer. Sequences were determined with the DNAman software analysis system (Lynnon Corporation, Quebec, Canada).

## Results

### Clinical data

**Figure 2:**
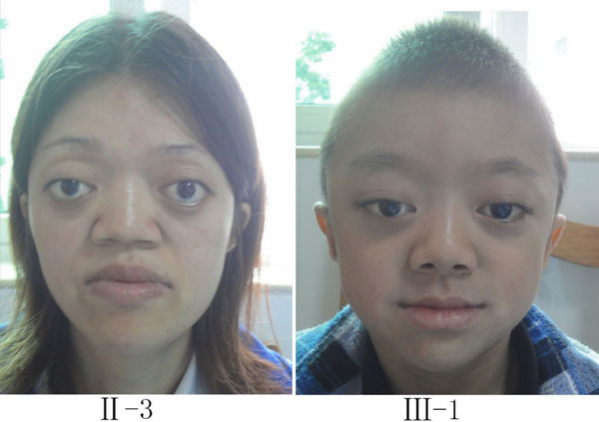
Photographs of II-3 (**A**) and III-1 (**B**). Both patients have ocular proptosis and midface hypoplasia.

**Figure 3:**
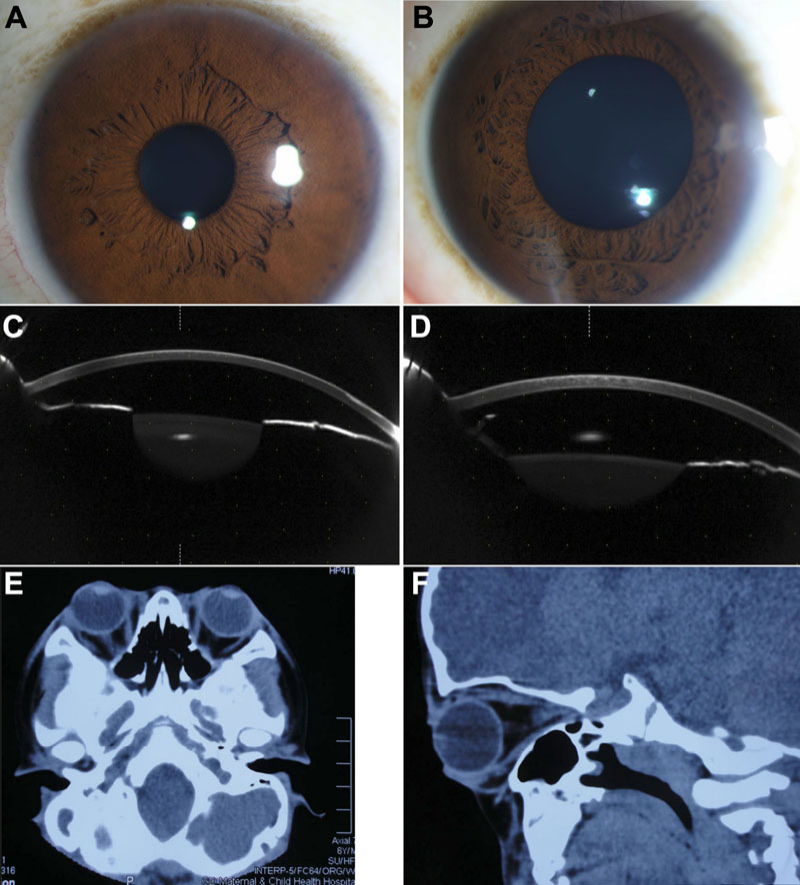
Examination results of II-3 and III-1. **A**: Anterior segment photograph of II-3. **B**: Anterior segment photograph of III-1. **C**: The anterior segment picture of II-3 by Pentacam. **D**: The anterior segment picture of III-1 by Pentacam. **E**, **F**: Shallow orbits and ocular proptosis of III-1 using computed tomography (CT).

The Chinese family studied in this report was from the southern area of China. Two individuals, in two successive generations, were found to have the same congenital disease (Figure 1). These patients had shallow orbits and ocular proptosis, accompanied by midface hypoplasia, craniosynostosis, a curved, beaklike nose, and clinically normal hands and feet. They have had normal vision since early childhood, but they displayed a surprised look (Figure 2). Applanation tonometry revealed normal intraocular pressures in both eyes of both patients. The corneas were normal in size and transparency, and the lenses were positioned normally and remained clear (Figure 3A,B).

The visual acuity, of the II-3 patient (30 years old, female) as measured by LogMAR, was 0.10 (OD) and 0.10 (OS). The axial lengths of II-3 were 21.99 mm (OD) and 22.13 mm (OS). No abnormalities were detected in the lenses, retinas, choroids, or optic nerves. The eye prominence of II-3 was 19 mm (OD) and 20 mm (OS). The anterior segment photograph is shown in Figure 3C—the anterior chamber depths were 2.41 mm (OD) and 2.47 mm (OS).

The visual acuity of the III-1 patient (7 years old, male) was 0.0 (OD) and 0.0 (OS). Axial lengths were 22.32 mm (OD) and 22.25 mm (OS). No abnormalities were detected in the lenses, retinas, choroids, or optic nerves. The anterior segment photograph is shown in Figure 3D; the anterior chamber depths were 2.57 mm (OD) and 2.64 mm (OS). The prominence of the III-1 was 17 mm (OD) and 16 mm (OS).

Computed tomography (CT) of the skull of III-1 (Figure 3E,F) was performed at 7 years of age, revealing shallow orbits.

### Mutation screening

**Figure 4:**
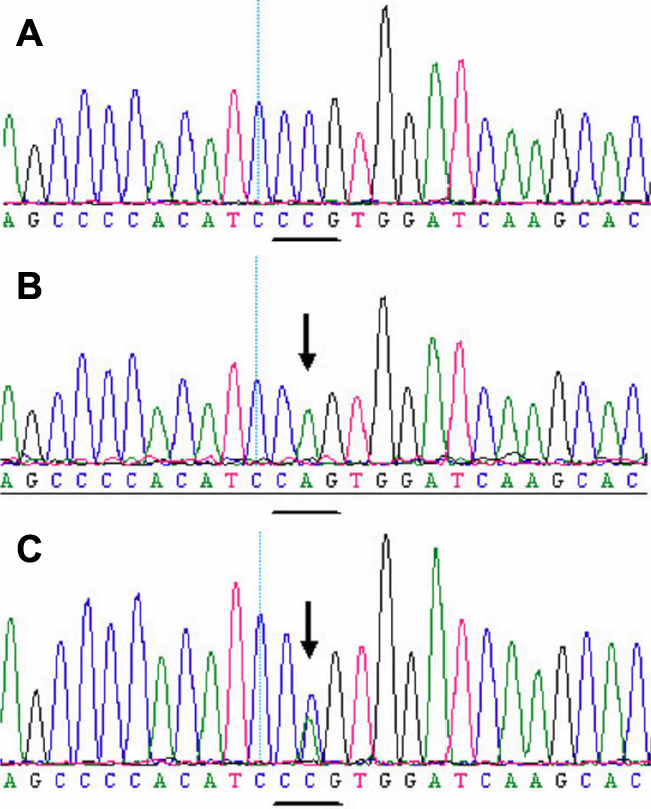
DNA sequence of a part of *FGF2* in the affected patients and unaffected individuals. **A**: A mutation c.866A>C (Gln289Pro) in exon 8 in the affected individuals. **B**: Sequence of the normal allele of exon 8 subcloned into the pGEM-T vector used as a control. **C**: A heterozygous missense mutation c.866A>C (Gln289Pro) in exon 8 in the affected individuals. The mutation causes the glutarnine 289 codon (CAG) to change to a proline codon (CCG).

A heterozygous missense mutation c.866A>C (Gln289Pro) in exon 8 (Figure 4) was identified in the two affected individuals, but not in any of the unaffected family members and the normal controls. The mutation causes the Glutarnine 289 codon (CAG) to change to a Proline codon (CCG).

## Discussion

In this study, we found one mutation in exon 8 of the *FGFR2* gene that is associated with Crouzon Syndrome: c.866A>C. This mutation, rather than a rare polymorphism in the normal population, is the causative mutation in the family.

The c.866A>C mutation (Gln289Pro) was identified for the ﬁrst time in *FGFR2* in Chinese patients; however, it occurred at a hotspot for mutations, which have been found in other ethnic groups [14].

Previously described mutations causing craniosynostosis are widely distributed across the FGFR2 protein, yet the majority localize in some amino acids that form the S-S bond in the IgIIIa/IIIc domain [14]. Severe phenotypes have been associated with mutations, causing loss or addition of cysteine residues, thereby resulting in the disruption of the protein’s structure, dimerization, and activation of the receptor [15].

Kress et al. [10] found that some mutations, such as Gln289Pro, Phe276Val, Ile288Ser, and Thr341Pro, affect conserved residues in the IgIII loop or at its margins, and may reduce the stability of the core loop domain, as suggested by three-dimensional modeling of the IgIII FGFR2 domain [16].

The ocular manifestations in the conditions caused by *FGFR* mutations included downslanting palpebral fissures, shallow orbits and proptosis, hypertelorism, and strabismus. Okajima et al. [17] reported that some patients with an *FGFR2* mutation had ocular anterior chamber dysgenesis, including Peters anomaly, optic nerve hypoplasia, scleralization of the cornea, and corectopia in craniosynostosis syndromes. Fortunately, the patients in our study did not have serious ocular disorders.

Actually, Chow et al. [18] and Robinson et al. [19] have already found that FGFRs may play an important role in regulating anteroposterior patterns of lens cell behavior. Transgenic expression of dominant-negative Fgfrs in the lenses of mice resulted in reduced fiber cell elongation. However, few patients with *FGFR* mutations had congenital cataracts, as in our study.

In summary, this study identified one novel mutation of *FGFR2* in a Chinese family with Crouzon syndrome. This finding expands the mutation spectrum of *FGFR2* and is useful and valuable for genetic counseling and prenatal diagnosis in families with Crouzon syndrome without serious ocular disorders.
